# Management of acute cancer-related pain in a patient with opioid use disorder: a case report

**DOI:** 10.1186/s13256-026-06261-y

**Published:** 2026-07-06

**Authors:** George Fawzy, James Mears, Lisa Calderwood

**Affiliations:** 1 Department of Internal Medicine, Charleston Area Medical Center, Charleston, WV 25304 USA; 2 Division Chief of Hospice and Palliative Care, Charleston Area Medical Center, West Virginia University, Charleston, WV 25304 USA; 3 CAMC Institute for Academic Medicine, Charleston, WV 25304 USA; 4 West Virginia University, Charleston Campus, Charleston, USA

**Keywords:** Cancer-pain management, Opioid use disorder, Palliative care

## Abstract

**Background:**

Managing cancer-related pain in patients with opioid use disorder (OUD) poses significant challenges. We present this case of an OUD patient with severe intractable pain from metastatic testicular cancer to illustrate one approach to effective pain management.

**Case presentation:**

A 31-year-old non-Hispanic White man with OUD experienced acute intractable cancer-related pain that was ultimately controlled with > 4700 daily oral morphine equivalents of patient-controlled fentanyl analgesia. He successfully weaned to oral methadone with oxycodone for breakthrough pain.

**Conclusions:**

Early palliative care involvement can facilitate effective pain control in patients with cancer and OUD.

## Background

Managing cancer-related pain in patients with concomitant opioid use disorder (OUD) has always been a challenge. According to a systematic literature review, about one-third of generalized cancer patients are still not prescribed pain medication that aligns with their reported pain severity [1]. Among patients already receiving opioid therapy, those on doses exceeding 400 oral morphine equivalents (OME) were found, in a 2018 retrospective study, to be at higher risk of inadequate pain treatment when presenting to the ED with acute cancer-related pain [2]. Patients with OUD often require higher morphine equivalents as compared with patients who are opioid-naïve to attain comparable levels of analgesia [3]. In addition, those already taking high OME often experienced opioid-induced hyperalgesia [4]. Guidelines from the American Society of Clinical Oncology recommend that patients with OUD and moderate-to-severe cancer-related pain or who are undergoing active treatment should be offered opioids, and that treating physicians should collaborate with a palliative care, pain, or substance use disorder (SUD) specialist to ensure the best course of action for pain management in patients with SUD [5].

One survey, exploring clinician barriers to effective opioid use in cancer-related pain treatment, suggests that healthcare professionals were primarily concerned about patients developing addiction, experiencing opioid-related adverse effects, and lacking sufficient education on opioid dosing and patient monitoring [6]. It also highlights considerable knowledge deficits among healthcare professionals related to opioid prescribing and pain management [6]. Another qualitative interview study identifies frequent challenges oncologists encounter when prescribing opioids [7]. Patients undergoing opioid therapy for cancer-related pain often experience stigma from healthcare providers and society at large [7]. In addition, fear of addiction can diminish patients’ willingness to accept prescribed opioids [7]. Furthermore, misinterpretation of safe and effective opioid prescribing guidelines frequently creates obstacles that limit patient access to these medications [7]. Proposed improvements include better education concerning the safety of opioids for cancer-related pain, and additional support from pain and/or palliative care specialists [7].

This case report details a patient with acute severe cancer-related pain who was opioid tolerant and required high OME for pain control. The case illustrates one approach to effective pain management and underscores the role of palliative care in achieving optimal pain control.

## Case presentation

**Fig. 1:**
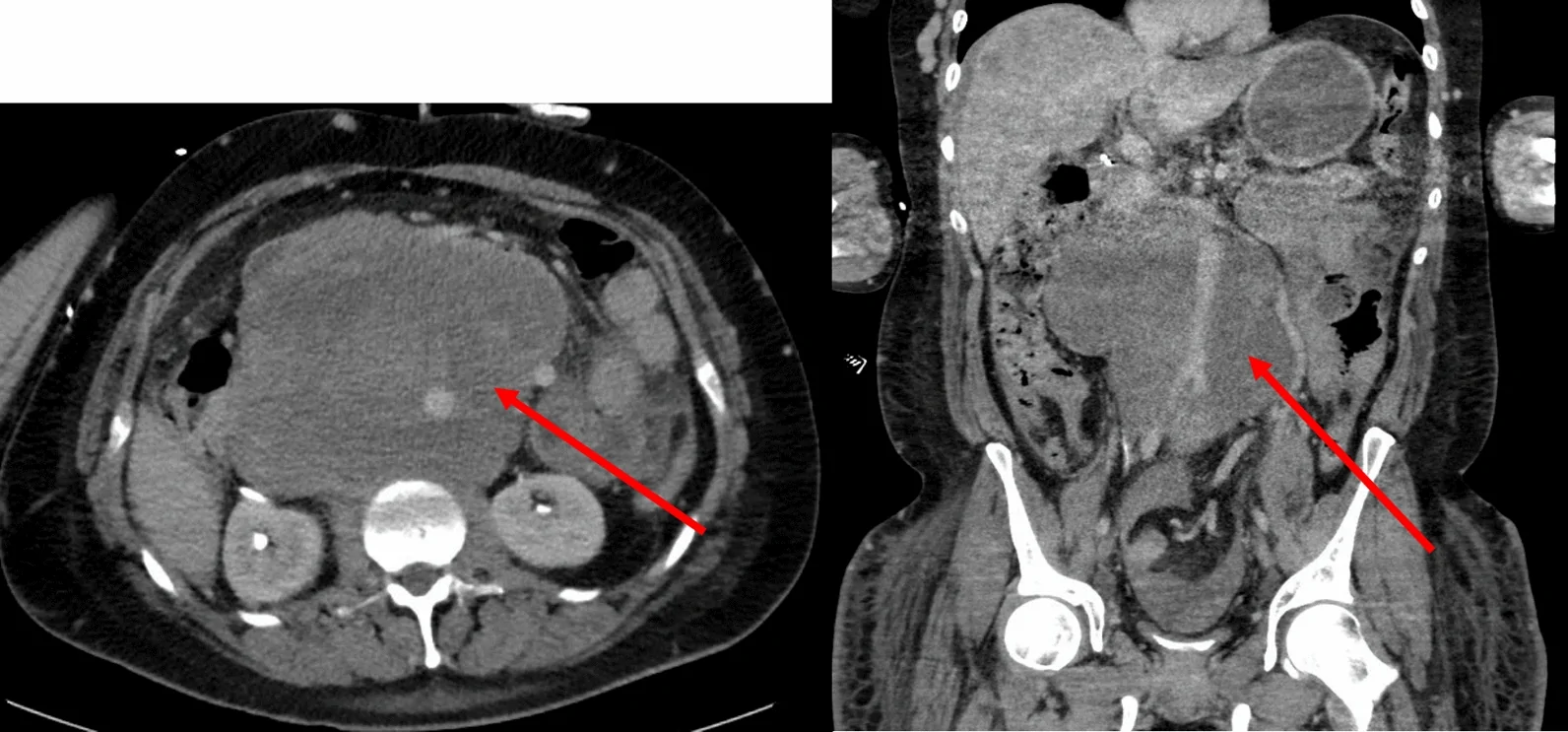
Metastatic retroperitoneal mass with complete encasement of the aorta and vascular structures (red arrows)

A 31-year-old non-Hispanic White man with no significant past medical history presented to the hospital due to diffuse acute intractable abdominal pain which had persisted for approximately 1 week. Associated symptoms included nausea and new-onset leg swelling bilaterally. The patient has no known relevant family history. He self-reported previous heroin use; he had been successful with a methadone maintenance program, using 110 mg per day, until about 1 month prior to admission when he had abdominal pain that was so severe that he resorted to using intranasal fentanyl (self-reporting use of up to 1 gm per day). Physical examination revealed a large, indurated, tender right testicular mass, distended abdomen, and bilateral leg edema (grade 2 +). Significant laboratory findings included elevated alpha-fetoprotein level (3529 ng/mL) and lactate dehydrogenase level (356 units/L). Computed tomography (CT) scan of his chest, abdomen and pelvis with contrast showed a 17 × 12 cm heterogenous retroperitoneal mass encasing his inferior vena cava and abdominal aorta (Fig. 1), numerous hepatic and pulmonary masses, and multiple enlarged inguinal lymph nodes consistent with metastatic disease. A biopsy of a retroperitoneal lymph node and right radical orchiectomy were performed, and the pathology findings were consistent with metastatic testicular mixed germ-cell cancer.

The patient had persistent intractable pain he described as “10/10 in intensity” despite the use of 20 mg IV hydromorphone and 60 mg of oral oxycodone within 24 h. It was later found that the patient was actively using illicit fentanyl (approximately 1 g per day) prior to admission. Palliative care was consulted to assist with pain management, and the peer SUD recovery team was asked to provide support with counseling and behavioral therapy. Due to escalating pain-management requirements, all oral opioids were discontinued, and he was started on a continuous intravenous hydromorphone patient-controlled analgesia (PCA) pump while receiving boluses of IV hydromorphone every 20 min as needed, which reduced his self-reported pain level to “6/10”. He was later transitioned to IV fentanyl via the PCA pump because of pharmacy shortage of IV hydromorphone and availability of IV fentanyl. The patient used a maximum of 28,722 mcg of IV fentanyl via the PCA by day 5 with settings of 600 mcg/h and 400 mcg every 20 min as needed. His opioid requirements eventually exceeded 4700 OME per day to achieve adequate pain control. He also received celiac plexus neurolysis but the patient did not get much relief with that intervention. He started chemotherapy in the hospital, which consisted of bleomycin, etoposide, and cisplatin. The patient was hospitalized for a total of 27 days, during which he was weaned off the fentanyl PCA to 40 mg methadone twice a day and 40 mg oxycodone every 4 h as needed for breakthrough pain at discharge with adequate control of pain and good functional status. He was discharged with close follow-up with his palliative care, oncology, and primary care providers. During multiple follow-up visits as an inpatient and as an outpatient, methadone was eventually titrated up to 55 mg twice a day, and the ECG remained stable with normal sinus rhythm and normal QTc intervals. Breakthrough oxycodone dose was reduced to 20 mg every 4 h as needed. The patient’s doses remained stable for the past year with good pain control, and medications were well-tolerated. Periodic urine drug screens were positive only for the prescribed methadone, oxycodone, and their metabolites. The state prescription drug monitoring program (PDMP) was monitored with each visit, and reports were consistent with his prescribed medications. The patient signed a controlled substance agreement and was adherent to that. The patient was functional initially without any undue consequences from the opioid regimen. However, later, he developed brain metastases and intracerebral hemorrhage causing left hemiparesis. He underwent radiation therapy. He also developed kidney injury from chemotherapy which has not recovered. He underwent physical and occupational therapy with some improvement of function. However, his functional status declined from an initial palliative performance scale of 70% down to a current level of 30–40%. He has significant help from family at home. Although much of the cancer and metastatic disease was dealt with by chemotherapy, some residual disease in the abdomen remained. Surgical consultation was performed, and it was felt that the patient would require an extensive surgical intervention with vascular reconstruction of intra-abdominal vessels which was going to be too challenging to undertake.

## Conclusions and discussion

This case highlights the complexity of acute pain control in an individual with OUD requiring high OMEs to achieve considerable pain control as compared with individuals who are opioid-naïve. His pain was ultimately managed with fentanyl administered using a PCA pump while transitioning to methadone to achieve adequate analgesia. OUD does not preclude utilizing opioids for cancer-pain analgesia, but clear guidelines to follow when managing cancer pain complicated by OUD are lacking [8]. With close attention to patient adherence to safe-use practices, regular follow-up, and ongoing monitoring, opioids should be prescribed based on clinical indication and tapered when appropriate [8]. The peer recovery team evaluated the patient during his hospitalization, highlighting that counseling and behavioral therapy remain essential components of OUD treatment, even when medication-assisted treatment (MAT) is employed. As part of the patient’s ongoing care, harm-reduction strategies will continue. In addition, the patient’s medications will be administered and monitored at home by his sister.

A matched case–control analysis demonstrated that patients admitted for acute cancer-related pain with OUD were 90% less likely to receive any dose escalation of their opioid regimen, irrespective of their OUD remission status [3]. However, following palliative medicine consultation, there were no differences in opioid administration observed between patients with and without a history of OUD, indicating that dose escalation was clinically appropriate for both groups [3]. In our patient, PCA using fentanyl was used for acute pain management, while methadone was used for long-term maintenance only after collaboration with palliative care. Calculations for opioid conversions were based on adapted recommendations from ML McPherson, 2018 and not based on reported use of fentanyl, since it is unknown how pure illicit fentanyl is and how much the patient is actually receiving [9]. Repeated frequent trials of hydromorphone boluses were used to gauge the initial settings for the hydromorphone PCA, then the reference table was used to convert from IV hydromorphone to IV fentanyl. For oral morphine equivalents over 200 mg or age greater than 65, McPherson recommends a ratio of 20:1 (morphine: methadone) but no more than 40 mg of methadone initially per day [9]. A PCA pump was used as it provides several benefits in the acute setting. PCA can help avoid multiple nursing requests and delays in receiving analgesia while experiencing breakthrough episodes of pain [10]. The PCA pump can also be titrated to the minimum effective plasma concentration needed to avoid the risk of overdose or inadequate analgesia and can aid in weaning to oral opioids [10].

In addition to utilization of full agonist opioids, adjunct therapies for cancer-related pain include buprenorphine–naltrexone, methadone, ketamine and interventional therapies [11]. However, high-quality data supporting some of these modalities is limited, and some are not always feasible due to institutional differences, clinician experience, and regulatory policies. For this patient, we determined that methadone would be the most appropriate option to address both the patient’s cancer-related pain and OUD. While methadone is not a first- or second-line treatment for cancer-pain management at some institutions, many observational studies demonstrate that it can be a feasible option as analgesia for refractory cancer pain and can help with addiction symptoms through dose escalation while being cost effective [12, 13]. In patients who have a favorable cancer prognosis, and if treatment is effective and remission is achieved, their care could include tapering opioids and continuing MAT through for their OUD. However, such an approach may not be applicable to patients with limited life expectancy due to cancer.

When managing pain in patients with a history of OUD, early involvement of pain specialists, including a palliative care or SUD specialist, can help provide adequate pain analgesia and high-quality care. More prospective studies are needed to develop a universally applicable approach to managing cancer-related pain in patients with SUDs. Future studies should aim to assist providers in preventing suboptimal treatment while minimizing the risk of opioid misuse.

## Data Availability

Not applicable.

## Abbreviations

CT: Computed tomography
ED: Emergency department
IV: Intravenous
MAT: Medication-assisted treatment
OME: Oral morphine equivalents
OUD: Opioid use disorder
PCA: Patient-controlled analgesia
SUD: Substance user disorder

## Declarations

### Declaration of AI Use

Artificial intelligence (AI) tools were used solely to assist with language editing, including grammar correction, improving clarify, and paraphrasing.

### Disclaimers

This manuscript is not currently under consideration for any other publication or presentation.
